# Investigation of the Interaction of Sodium Chloride and Two Amino Sulfonic Acids, HEPES and MOPSO, by EMF Measurements

**DOI:** 10.6028/jres.096.049

**Published:** 1991

**Authors:** Y. C. Wu, Darning Feng, W. F. Koch

**Affiliations:** National Institute of Standards and Technology, Gaithersburg, MD 20899

**Keywords:** activity coefficient, amino acid, emf, HEPES, MOPSO, salt interaction, sodium chloride

## Abstract

Ionic interactions in the two systems NaCl-HEPES (N-2-hydroxyethylpiperazine-N′-2-ethanesulfonic acid) and NaCl-MOPSO (3-(N-Morpholino)-2-hydroxypropanesulfonic acid) have been studied in terms of their mutual influence on the respective activity coefficients of each component. Activity coefficients for each component of the two systems and for corresponding buffers are calculated from emf measurements of solutions containing NaCl, the aminosulfonic acid, and its conjugate base in a NalSE/solution/AgCl-Ag cell at 5, 15, 25, and 37 °C.

## 1. Introduction

The interaction between an amino acid and a neutral salt in solution can be described in terms of the activity coefficients of the components [1]. In this laboratory, when we decided to establish HEPES(N-2-hydroxyethylpiperazine-N′-2-ethane-sulfonic acid) as a pH Standard Reference Material (SRM) for physiological application, we needed to know the effect of HEPES on the activity coefficient of NaCl. Since there were no relevant data in the literature, we had to assume that the influence of HEPES on the activity coefficient of NaCl was the same as that of glycine on NaCl [2]. Even though the effect was small, it was necessary to verify our assumption. MOPSO(3-[N-Morpholino]-2-hydroxypropanesulfonic acid) is another candidate for an SRM in the physiological pH range. The influence of MOPSO on the activity coefficient of NaCl is also not known. The purposes of this investigation were to determine the mutual influence between NaCl and HEPES, and between NaCl and MOPSO, and to provide the data needed to calculate the activity coefficients for NaCl, HEPES, and MOPSO in the respective mixed solutions. The determination of the activity coefficient of amino acids and NaCl in their mixed solutions may be done by potentiometry. One approach using a sodium amalgam electrode has been reported [3]. However, it was found that the amino acids decomposed sodium amalgam and evolution of hydrogen gas was observed. For this reason, the sodium ion-selective electrode (NalSE) provided a more convenient approach to this type of determination.

## 2. Experimental

### 2.1 The Apparatus

The cells studied were of the type

$$\mathrm{NaISE}/\mathrm{NaCl}\left(m_{1}\right)/\text{AgCl-Ag}$$ (I)

for the pure NaCl solution, and

$$\begin{array}{c} \mathrm{NaISE}/\mathrm{NaCl}\left(m_{1}\right),\mathrm{HEPES}\left(m_{2}\right),\\ \text{NaHEPESate}\left(m_{3}\right)/\text{AgCl-Ag} \end{array}$$ (II)

$$\begin{array}{c} \mathrm{NaISE}/\mathrm{NaCl}\left(m_{1}\right),\mathrm{MOPSO}\left(m_{2}\right),\\ \mathrm{NaMOPSOate}\left(m_{3}\right)/\text{AgCl-Ag} \end{array}$$ (III)

for the two-component systems (*m*_3_ *= 0*) and three-component systems for HEPES (II) and MOPSO (III). A glass NaISE (13-620-500, Fisher Scientific)^1^ was used for the measurements for solutions containing HEPES. However, in solutions containing MOPSO the emf readings drifted continually and were not reproducible. It appeared that the MOPSO coated the glass surface of the electrode. Thus, it was necessary to use a PVC membrane neutral carrier-based NalSE (provided by Prof. W. Simon, Zürich, Switzerland [4]) for the MOPSO system. The silver-silver chloride reference electrodes were identical with those used previously in this laboratory [2]. emfs were measured using an Orion 701A pH meter with a sensitivity of 0.1 mV. Measurements were made at various temperatures in a bath controlled to ±0.01 °C [2]. The electrodes were calibrated daily using NaCl solutions in cell (I). Activity coefficients of NaCl were taken from the literature [5]. The observed (Nernstian) slopes *k* for both glass and PVC membrane NaISEs were constant for each calibration with a standard deviation of 0.05 mV for the whole range of temperatures in this study. The values are shown in Tables 1 and 2. They were about 1 mV lower than the theoretical Nernstian slopes; hence, the electrodes were not thermodynamically reversible, but were still useful for the purpose of this study. The selectivity of the two NalSEs toward the hydrogen ion requires that the pH of the solutions should be between 4.5 and 10 at the NaCl concentration of 0.01–0.1 *m* [4,6]. In this study, the pH of the solutions was 5.5–7.5, which was well within the range of requirement.

### 2.2 Materials

NaCl was ACS reagent grade and was dried at 110 °C for 4 h before use. HEPES and MOPSO (Sigma Co., St. Louis, MO) were recrystallized from 80% and 70% ethanol/water solutions respectively, and were dried in a vacuum oven at 50 °C overnight. Their sodium salt solutions were made by neutralizing the acid solutions with standard sodium hydroxide. Stock solutions were prepared by weight. All mass measurements were made with an accuracy to 0.03 mass percent and air buoyancy corrections were applied for all masses used. The laboratory distilled water used in this experiment was passed through a deionizing column and had a conductivity of less than 1 µS/cm.

## 3. Results

The emfs of cells (II) and (III) can be empirically expressed as an equation similar to the Nernstian equation:

$$E=E_{0}-k\log\left(m_{\mathrm{Na}}m_{\mathrm{Cl}}{\gamma_{1}}^{2}\right)$$ (1)

where *γ*_1_ is the mean molai activity coefficient of NaCl and *m* is the molai concentration, *E*_0_ is an extrapolated constant, and *k* (in mV) is another constant.

For pure NaCl solutions in cell (I), the emf is expressed as

$$E_{p}=E_{0}-2k\;\log\left(m_{1}\gamma_{1}{}^{\circ}\right)$$ (2)

where *γ*_1_° denotes the mean molai activity coefficient for pure NaCl solution. *E*_0_ and *k* can be obtained from this equation by using a least square fitting procedure. For the HEPES-NaCl and MOPSO-NaCl two-component systems, if the concentrations of NaCl in cells (II) and (III) are the same as that in the pure NaCl solutions, then we can combine Eqs. (1) and (2) to obtain

$$\log\left(\gamma_{1}/{\gamma_{1}}^{o}\right)=\left(E_{p}-E\right)/2k$$ (3)

where *E*_p_ is the emf for pure NaCl solution from cell (I). All the emfs and log(*γ*_1_/*γ*_1_°) values are listed in Tables 1 and 2 together with *k* values at four temperatures. All the molalities in Tables 1 and 2 are accurate to better than 0.1%.

### 3.1 Influence of HEPES and MOPSO on the Activity Coefficient of NaCl

**Figure 1:**
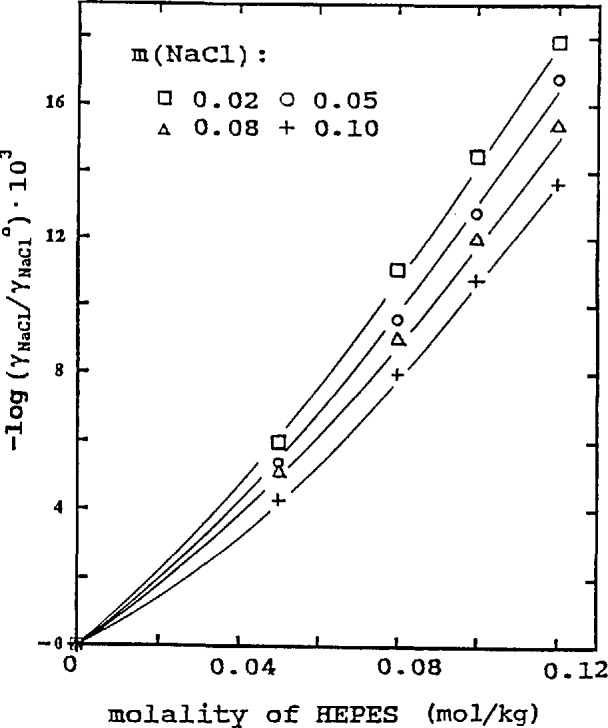
Influence of HEPES on activity coefficient of NaCl at 25 °C.

**Figure 2:**
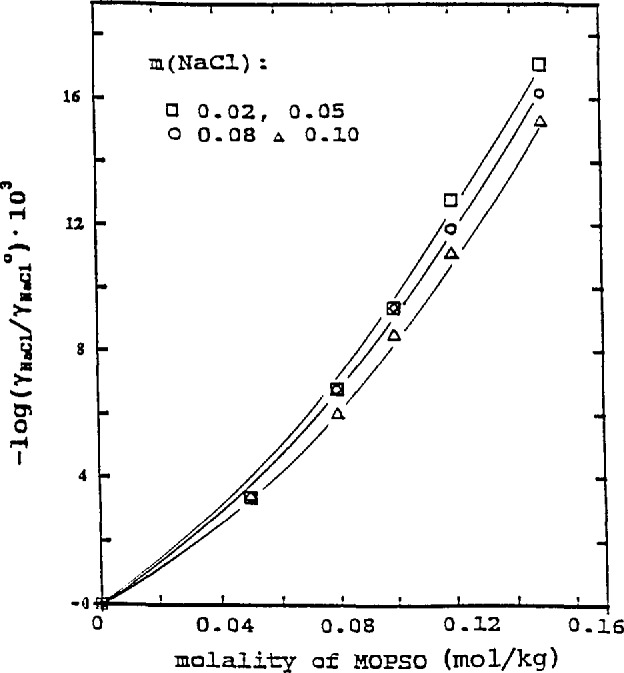
Influence of MOPSO on activity coefficient of NaCl at 25 °C.

The values of log(*γ*_1_/*γ*_1_°) at 25 °C from Tables 1 and 2 were plotted against the molality of HEPES and MOPSO in Figs. 1 and 2, respectively. The trend of the behavior of log(*γ*_1_/*γ*_1_°) was similar to that for other amino acids [1], HEPES and MOPSO diminished the activity coefficients of NaCl; the effect of HEPES was more pronounced than that of MOPSO. An increase in the NaCl concentration decreased the effect of amino acids. The values of log(*γ*_1_/*γ*_1_°) at 25 °C can be represented by the following equations:

$$\begin{array}{l} \text{HEPES-NaCl solution},\\ \;-\log\left(\gamma_{1}/\gamma_{1}{}^{\circ}\right)=\left(0.11-0.42m_{1}\right)m_{2}\\ \;+\left(0.45-0.44m_{1}\right){m_{2}}^{2} \end{array}$$ (4)

$$\begin{array}{l} \text{MOPSO-NaCl solution},\\ \;-\log\left(\gamma_{1}/\gamma_{1}{}^{\circ}\right)=\left(0.05+0.04m_{1}\right)m_{2}\\ \;+\left(0.51-1.4m_{1}\right){m_{2}}^{2}. \end{array}$$ (5)

**Figure 3:**
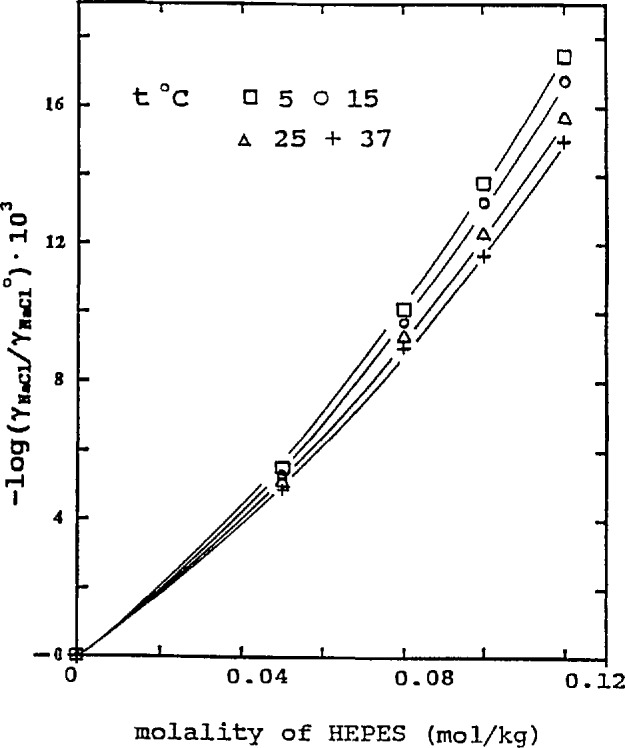
Influence of HEPES on activity coefficient of NaCl at different temperatures, *m* (NaCl) = 0.08.

**Figure 4:**
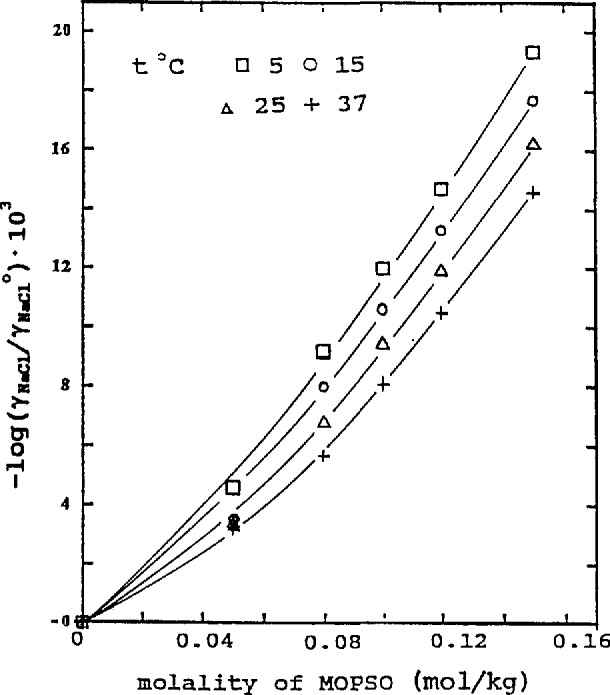
Influence of MOPSO on activity coefficient of NaCl at different temperatures, *m* (NaCl) = 0.08.

The temperature effect is shown in Figs. 3 and 4 at *m*_2_ = 0.08. In our previous paper [2], we used the following equation for HEPES-NaCl solutions at *m*_NaCl_ = 0.08 and assumed that it was temperature-independent:

$$-\log\left(\gamma_{1}/\gamma_{1}{}^{\circ}\right)=0.125\;m_{\mathrm{HEPES}}.$$ (6)

The log(*γ*_1_/*γ*_1_°) values calculated from Eq. (6) and observed experimentally (cf. Table 1) are listed in Table 3. The difference is within the experimental uncertainty.

### 3.2 The Activity Coefficient of NaCl in MOPSO-NaMOPSOate-NaCl Buffer Solutions

Data for the activity coefficients of NaCl are needed in the determination of the pH of three-component MOPSO-NaMOPSOate-NaCl buffer solutions. In this work, log *γ*_NaCl_ in 0.05 and 0.08 *m* equimolal MOPSO-NaMOPSOate-NaCl solutions was directly determined by using cell (III). For this cell,

$$\log\gamma_{1}=1/2\left[\left(E_{0}-E\right)/k-\log\left(m_{1}+m_{3}\right)m_{1}\right]$$ (7)

where *E*_0_ and *k* were obtained from the measurements of cell (I). The results, listed in Table 4, are used for the determination of the pH values in these buffer solutions.

### 3.3 Activity Coefficients of HEPES and MOPSO

According to the Gibbs-Duhem equation, the activity coefficient of an amino acid in an amino acid-NaCl two-component solution can be calculated from the activity coefficient of NaCl in the same solution [1]:

$$\log\left(\gamma_{2}/\gamma_{2}{}^{\circ}\right)=2\int_{0}^{m_{1}}\partial \log\gamma_{1}/\partial m_{2})\partial m_{1}$$ (8)

where *γ*_2_ is the activity coefficient of the amino acid in the mixed solution and *γ*_2_° is that for an isomolal solution without NaCl, *γ*_1_ is the activity coefficient of NaCl in this mixed solution. By substituting Eqs. (4) and (5) into Eq. (8), log(*γ*_2_/*γ*_2_°) can be evaluated, log*γ*_2_° for HEPES at 25 °C was calculated from emf measurements of the following cell,

$$H_{2}\left(g,1\;\text{atm}\right)/\mathrm{HEPES},\mathrm{NaHEPESate},\mathrm{NaCl}/\mathrm{AgCl},\mathrm{Ag}\left(s\right)$$ (IV)

as described in our previous paper [2], in which *γz±* represented *γ*_HEPES_:

$$-\log\gamma_{\mathrm{HEPES}}{}^{\circ}=0.20m_{\mathrm{HEPES}}.$$ (9)

Analogous measurements were carried out for MOPSO at 25 °C [7] and the following result was obtained:

$$-\log\gamma_{\mathrm{MOPSO}}{}^{\circ}=0.10m_{\mathrm{MOPSO}}.$$ (10)

Thus, from Eqs. (8), (9) and (10), the activity coefficients of HEPES and MOPSO at 25 °C are expressed as follows:

$$\begin{array}{l} \log\gamma_{\mathrm{HEPES}}=\left(-0.22m_{\mathrm{NaCl}}+0.42{m_{\mathrm{NaCl}}}^{2}\right)\\ -\left(0.20+1.8m_{\mathrm{NaCl}}-0.88{m_{\mathrm{NaCl}}}^{2}\right)m_{\mathrm{HEPES}} \end{array}$$ (11)

$$\begin{array}{l} \log\gamma_{\mathrm{MOPSO}}=\left(-0.10m_{\mathrm{NaCl}}-0.04{m_{\mathrm{NaCl}}}^{2}\right)\\ -\left(0.10+2.0m_{\mathrm{NaCl}}-2.8{m_{\mathrm{NaCl}}}^{2}\right)m_{\mathrm{MOPSO}} \end{array}$$ (12)

Taking the first-order terms as an approximation, the following simpler forms result. For comparison, the analogous expression for glycine [1] is also shown:

$$\log\gamma_{\mathrm{HEPES}}=-0.20m_{\mathrm{HEPES}}-0.22m_{\mathrm{NaCl}}$$ (13)

$$\log\gamma_{\mathrm{MOPSO}}=-0.10m_{\mathrm{MOPSO}}-0.10m_{\mathrm{NaCl}}$$ (14)

$$\log\gamma_{\text{Glycine}}=-0.10m_{\text{Glycine}}-0.28m_{\mathrm{NaCl}}.$$ (15)

## 4. Discussion

All the parameters used for Eqs. (4), (5), (13), and (14) are empirical. They are derived from the least square fitting of the experimental data and are only valid within the range of the concentrations in this work. According to Cohn and Edsall [1], at low NaCl concentrations, most amino acids diminish the activity coefficients of NaCl, and NaCl in turn diminishes the activity coefficients of amino acids. The present work demonstrates a similar phenomenon in the NaCl-HEPES and NaCl-MOPSO systems.

Because of the relatively high input impedance of the NaISE, a pH meter was used to measure the emfs of cells (I), (II), and (III). The resolution of this pH meter was 0.1 mV. This corresponds to an uncertainty of 0.0017 in log(*γ*_1_/*γ*_1_°). Each emf value reported in Tables 1 and 2 represents the mean of two separate measurements. The differences between each pair of emf values never exceeded 0.1 mV. Therefore, the overall uncertainty of log(*γ*_1_/*γ*_1_°) is estimated to be $0.0017/\sqrt{2}=0.0012$. The trend of the influence of the amino acid on the activity coefficients of NaCl, as indicated by log(*γ*_1_/*γ*_1_°) and shown in Figs. 1–4, is more evident at higher concentrations of the two amino acids. As the concentration becomes lower, the influence diminishes. In the lowest concentration region, the differences in the lg(*γ*_1_/*γ*_1_°) values at different NaCl concentrations or temperatures are within the limit of uncertainty and in some cases the log(*γ*_1_/*γ*_1_°) values practically overlap. The curves drawn in this region are merely to show the trend of the influence.

## Figures and Tables

**Table 1:** The emfs of the cell: NaISE/NaCl(*m*_1_), HEPES(*m*_2_), NaHEPESate(*m*_3_)/AgCl,Ag and the log(*γ*_1_/*γ*_1_°) values

*t*°C			5		15		25		37	
*m*_1_	*m*_2_	*m*_3_	*E*, mV	−log(*γ*_1_/*γ*_1_°)	*E*	−log(*γ*_1_/*γ*_1_°)	*E*	−log(*γ*_1_/*γ*_1_°)	*E*	−log(*γ*_1_/*γ*_1_°)
0.01	0	0	58.3		68.6		76.3		87.2	
0.02	0	0	27.7		36.3		42.8		52.1	
0.02	0.05	0					43.5	0.0060		
0.02	0.08	0					44.1	0.0111		
0.02	0.1	0					44.5	0.0145		
0.02	0.12	0					44.9	0.0179		
0.05	0	0	−13.1		−6.1		−1.0		6.9	
0.05	0.05	0					−0.4	0.0051		
0.05	0.08	0					0.2	0.0102		
0.05	0.1	0					0.5	0.0128		
0.05	0.12	0					1.0	0.0171		
0.08	0	0	−34.1		−27.6		−22.7		−16.3	
0.08	0.05	0	−33.5	0.0055	−27.0	0.0053	−22.1	0.0051	−15.7	0.0049
0.08	0.08	0	−33.0	0.0101	−26.5	0.0097	−21.6	0.0094	−15.2	0.0090
0.08	0.1	0	−32.6	0.0138	−26.1	0.0132	−21.3	0.0120	−14.9	0.0115
0.08	0.12	0	−32.2	0.0175	−25.7	0.0167	−20.9	0.0154	−14.6	0.0139
0.1	0	0	−42.6		−37.4		−33.3		−27.3	
0.1	0.05	0					−32.8	0.0043		
0.1	0.08	0					−32.3	0.0085		
0.1	0.1	0					−32.0	0.0111		
0.1	0.12	0					−31.7	0.0137		
0.08	0.08	0.08	−47.1		−41.1		−36.3		−30.1	

*k* (mV)			54.2		56.7		58.5		61.2	

**Table 2:** The emfe of the cell: NaISE/NaCl(*m*_1_), MOPSO(*m*_2_), NaMOPSOate(*m*_3_)/AgCl,Ag and the log(*γ*_1_/*γ*_1_°) values

*t*°C			5		15		25		37	
*m*_1_	*m*_2_	*m*_3_	*E*, mV	−log(*γ*_1_/*γ*_1_°)	*E*	−log(*γ*_1_/*γ*_1_°)	*E*	−log(*γ*_1_/*γ*_1_°)	*E*	−log(*γ*_1_/*γ*_1_°)
0.01	0	0	−19.8		−10.0		−2.5		10.5	
0.02	0	0	−51.2		−43.2		−36.0		−24.9	
0.02	0.05	0					−35.6	0.0034		
0.02	0.08	0					−35.2	0.0068		
0.02	0.1	0					−34.9	0.0094		
0.02	0.12	0					−34.5	0.0128		
0.02	0.15	0					−34.0	0.0171		
0.05	0	0	−91.8		−85.4		−79.5		−70.6	
0.05	0.05	0					−79.1	0.0034		
0.05	0.08	0					−78.7	0.0068		
0.05	0.1	0					−78.4	0.0094		
0.05	0.12	0					−78.0	0.0128		
0.05	0.15	0					−77.5	0.0171		
0.08	0	0	−112.7		−106.9		−101.8		−93.7	
0.08	0.05	0	−112.2	0.0046	−106.5	0.0035	−101.4	0.0034	−93.3	0.0032
0.08	0.08	0	−111.7	0.0092	−106.0	0.0080	−101.0	0.0068	−93.0	0.0057
0.08	0.1	0	−111.4	0.0120	−105.7	0.0106	−100.7	0.0094	−92.7	0.0081
0.08	0.12	0	−111.1	0.0147	−105.4	0.0133	−100.4	0.0119	−92.4	0.0105
0.08	0.15	0	−110.6	0.0193	−104.9	0.0177	−99.9	0.0162	−91.9	0.0146
0.1	0	0	−121.3		−116.8		−112.2		−105.2	
0.1	0.05	0					−111.8	0.0034		
0.1	0.08	0					−111.5	0.0060		
0.1	0.1	0					−111.2	0.0085		
0.1	0.12	0					−110.9	0.0111		
0.1	0.15	0					−110.4	0.0153		
0.05	0.05	0.05	−112.8		−104.0		−96.0		−83.7	
0.08	0.08	0.08	−132.2		−124.7		−117.2		−106.3	

*k*(mV)			54.4		57.0		58.6		61.7	

**Table 3:** Comparison of log(*γ*_1_/*γ*_1_°) at *m*_2_ = 0.08

*t* °C	−log(*γ*_1_/*γ*_1_°)	
	Observed	Calculated from Eq. (6)
5	0.0101	0.010
15	0.0097	0.010
25	0.0094	0.010
37	0.0090	0.010

**Table 4:** Determination of *γ*_NaCl_ in MOPSO-NaMOPSOate-NaCl solutions

*t* °C	−logγ_NaCl_	
	0.05 *m* (equimolal)	0.08 *m* (equimolal)
5	0.039	0.064
15	0.070	0.091
25	0.096	0.119
37	0.132	0.153

## Footnotes

1 Certain commercial equipment, instruments, or materials are identified in this report to specify adequately the experimental procedure. Such identification does not imply recommendation or endorsement by the National Institute of Standards and Technology, nor does it imply that the materials or equipment identified are necessarily the best available for the purpose.
